# Spatial and Feature-selective Attention Have Distinct, Interacting Effects on Population-level Tuning

**DOI:** 10.1162/jocn_a_01796

**Published:** 2022-01-05

**Authors:** Erin Goddard, Thomas A. Carlson, Alexandra Woolgar

**Affiliations:** 1 University of New South Wales; 2 Macquarie University, Sydney, New South Wales, Australia; 3 University of Sydney; 4 University of Cambridge

## Abstract

Attention can be deployed in different ways: When searching for a taxi in New York City, we can decide where to attend (e.g., to the street) and what to attend to (e.g., yellow cars). Although we use the same word to describe both processes, nonhuman primate data suggest that these produce distinct effects on neural tuning. This has been challenging to assess in humans, but here we used an opportunity afforded by multivariate decoding of MEG data. We found that attending to an object at a particular location and attending to a particular object feature produced effects that interacted multiplicatively. The two types of attention induced distinct patterns of enhancement in occipital cortex, with feature-selective attention producing relatively more enhancement of small feature differences and spatial attention producing relatively larger effects for larger feature differences. An information flow analysis further showed that stimulus representations in occipital cortex were Granger-caused by coding in frontal cortices earlier in time and that the timing of this feedback matched the onset of attention effects. The data suggest that spatial and feature-selective attention rely on distinct neural mechanisms that arise from frontal-occipital information exchange, interacting multiplicatively to selectively enhance task-relevant information.

## Introduction

At any moment, there is far more information available from our senses than we can process at once. Accordingly, only a subset of the available information is processed to a high level, making it crucial that the brain dynamically redistributes processing resources—selectively attends—to the most relevant information. We can deploy our attentional resources in different ways. For example, we can decide where to attend (e.g., to the item on the left, rather than the one on the right), and we can control what feature of that item we attend to (e.g., its color, rather than its shape). This allows us to dramatically reduce the computational requirements of our neural system and focus on the information that is most pertinent to our current task.

Each of these types of attention can change behavior, improving performance related to the attended location or stimulus feature (Carrasco, 2011; Pestilli & Carrasco, 2005; Saenz, Buracas, & Boynton, 2003; Rossi & Paradiso, 1995). Shifts in attention also induce neural changes consistent with greater neural resources being directed to representing attended over unattended information. These changes include shifts in the responses of individual neurons (Maunsell, 2015; Sprague, Saproo, & Serences, 2015; Reynolds & Heeger, 2009), changes in the overall responsiveness of brain regions (Gouws et al., 2014; Lennert, Cipriani, Jolicoeur, Cheyne, & Martinez-Trujillo, 2011; Serences & Boynton, 2007; Saenz et al., 2003; Saenz, Buracas, & Boynton, 2002; Chawla, Rees, & Friston, 1999; Corbetta, Miezin, Dobmeyer, Shulman, & Petersen, 1990), and changes in the information carried by a population response (Vaziri-Pashkam & Xu, 2017; Guggenmos et al., 2015; Woolgar, Williams, & Rich, 2015).

However, neuroimaging and electrophysiological studies have often focused on attention directed in space, or to a particular feature, in separate studies, making it difficult to predict how the different types of attention interact. Characterizing how different attentional mechanisms interact is essential, however, because they rarely operate in isolation. So far, results are mixed. Attending to a particular feature (e.g., red) changes baseline activity in the absence of visual stimuli (Chawla et al., 1999; Corbetta et al., 1990) and changes single-unit (McAdams & Maunsell, 2000) and neuronal population (Bartsch, Donohue, Strumpf, Schoenfeld, & Hopf, 2018; Cohen & Maunsell, 2011; Jehee, Brady, & Tong, 2011) responses across the visual field. The spatially diffuse effects of feature-based attention are also supported by results from visual search, where feature-based attention facilitates the parallel identification of items matching a target feature value (e.g., Peelen & Kastner, 2014; Bichot, Rossi, & Desimone, 2005). Conversely, attending to an object at a particular location can boost processing of all its features (Haazebroek, Raffone, & Hommel, 2017; O’Craven, Downing, & Kanwisher, 1999; Duncan, Humphreys, & Ward, 1997; Duncan, 1984). This is consistent with results showing these two subtypes of attention have largely independent, additive effects (Hayden & Gallant, 2009; Patzwahl & Treue, 2009; Treue & Martinez-Trujillo, 1999). Alternatively, others have reported more complex interactions, where the attended feature of an attended object was selectively enhanced whereas other information at that location was not (e.g., Jehee et al., 2011, and small multiplicative interaction in Hayden & Gallant, 2009).

The lack of human neuroimaging studies manipulating both types of attention also limits our ability to directly compare the effects of each. In nonhuman primates, substantial work suggests distinct effects of different attentional strategies on the tuning of individual neurons (e.g., reviewed by Reynolds & Heeger, 2009). However, stimulus information carried by large-scale neuronal populations cannot be trivially predicted from tuning curve properties alone, because factors such as response variance and correlation can significantly impact population information coding (Sprague et al., 2015; Moreno-Bote et al., 2014). Suggestive data from recent MEG/EEG studies indicate that these effects on tuning curves can be used to account for population-level responses in humans for feature-based (Bartsch et al., 2017; Störmer & Alvarez, 2014) and spatial (Foster, Thyer, Wennberg, & Awh, 2021) attention, but a direct contrast is needed to compare the population-level changes induced by each type of attention.

A final question concerns the top−down mechanisms supporting selection of information. The pFC (among other regions) is strongly implicated as a source of attentional control (Duncan, 2013; Miller & Cohen, 2001; Dehaene, Kerszberg, & Changeux, 1998), but the content of top−down signals is unclear. They may contain task-level information, including maps of attentional priority (Moore, Armstrong, & Fallah, 2003; Wolfe, 1994; Koch & Ullman, 1985), and the participant’s criterion (Luo & Maunsell, 2018). Alternatively, because frontoparietal control regions prioritize coding of task-relevant stimulus information (Jackson & Woolgar, 2018; Jackson, Rich, Williams, & Woolgar, 2017; Freedman & Assad, 2016; Woolgar et al., 2015; Freedman, Riesenhuber, Poggio, & Miller, 2001), they may bias processing elsewhere (Duncan, 2006; Desimone & Duncan, 1995) by transferring a representation of the attended information itself (see also Bichot, Heard, DeGennaro, & Desimone, 2015; Liu, Hospadaruk, Zhu, & Gardner, 2011).

Here, we used multivariate decoding of human MEG data, together with information flow analysis, to address these questions. We found strong multiplicative effects of where participants attended (left or right) and what they attended to (shape or color) on stimulus-related information coding in both frontal and occipital cortices. We further showed that the two attentional subtypes induced different effects on the population response, with distinct patterns of enhancement across feature differences of increasing magnitude. Finally, our information flow analysis tracked the exchange of information between frontal and occipital cortices over time. We found that the dominant direction of information flow was initially feedforward (from visual to frontal cortex) but later reversed and that the onset of this reversal corresponded to the strongest attentional modulation in the occipital lobe. We conclude that these two subtypes of attention have demonstrably different effects on population-level tuning in the human brain and interact multiplicatively to bring about selective focus on task-relevant information. The effects appear to be mediated by frontal-to-occipital exchange of item-specific, task-relevant stimulus information.

## Methods

### Participants

Participants’ (*n* = 20; 14 women, 6 men) ages ranged from 18 to 32 years (mean = 22.4 years), and they were each paid $50 as compensation for their time. All were righthanded, had normal or corrected-to-normal vision, had no history of neurological or psychiatric disorder, and were naïve to the purposes of the study. Participant recruitment and the experiment were conducted with the approval of the Macquarie University Human Research Ethics Committee.

### Visual Stimuli

**Figure 1:**
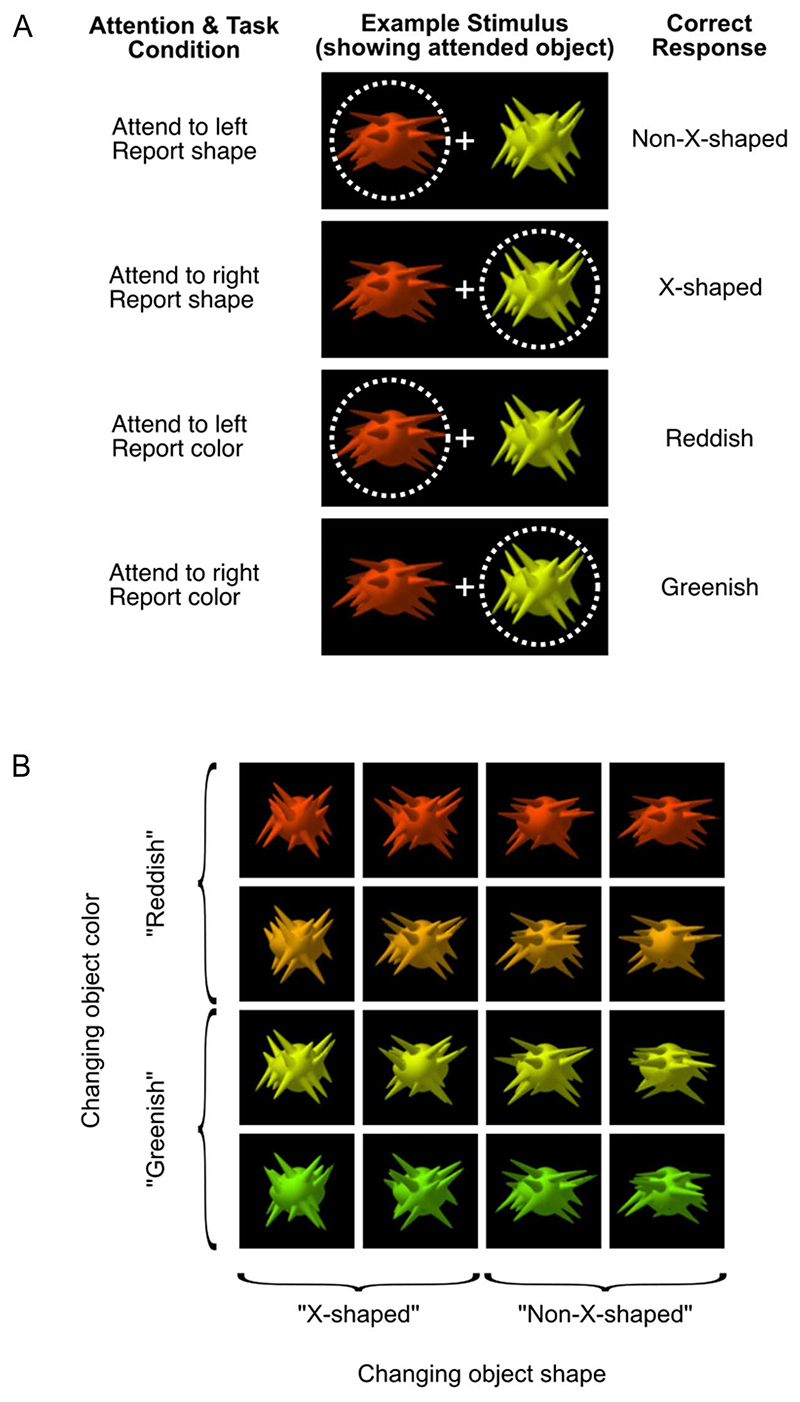
Visual stimuli showing task conditions (A) and stimulus dimensions (B). (A) Task conditions. At the start of each block of trials, participants were told the location to which they should direct their attention (left or right of fixation) and whether they should report the target object’s shape (“X-shaped” or “non-X-shaped”) or color (reddish or greenish). Two objects appeared on each trial, and participants covertly attended to one while we used eye tracking to monitor their fixation. The example illustrates how the same stimulus configuration was used in each of the four task conditions. The dotted circle indicates the location of spatial attention and was not visible during the experiment. (B) Stimulus dimensions. Each object varies systematically along two dimensions, color and shape. Participants categorized the attended object as either “greenish” or “reddish” (when reporting color) or as “X-shaped” or “non-X-shaped” (when reporting shape). On each trial, the objects were randomly selected from 100 exemplars with the same shape statistics but random variation in the location, length, and orientation of the spikes. This variability is illustrated in the shape variation between objects in the same column.

Visual stimuli were generated and presented using MATLAB (Version R2014b) and routines from Psychtoolbox (Kleiner, Brainard, & Pelli, 2007; Brainard, 1997; Pelli, 1997). We created novel object stimuli that varied in color and in their shape statistics (see Figure 1B) using custom code. The shapes were variants of “spikie” stimuli used in previous work (Jackson et al., 2017; Woolgar et al., 2015; Op de Beeck, Baker, DiCarlo, & Kanwisher, 2006), presented on a black background. We varied the spike orientation statistics to create four classes of “spikie” objects: strongly or weakly “X-shaped,” and strongly or weakly “non-X-shaped” (Figure 1B). In the shape-based task, participants categorized the target as “X-shaped” or “non-X-shaped.” We created 100 unique versions of each shape class by adding random variation in the spike locations, lengths, and orientations to ensure that no single feature was diagnostic of category and to encourage attention to the object’s overall shape.

In color, there were also four classes (strongly or weakly red, and strongly or weakly green; Figure 1B), which participants categorized as either “reddish” or “greenish” in the color task. Each object had a maximum luminance of 108.1 cd/m^2^ and constant *u*′*v*′ and *xy* chromaticity coordinates (Wyszecki & Stiles, 1982), which were as follows: strongly red *u*′*v*′: 0.35, 0.53 (*xy*: 0.56,0.38); weakly red *u*′*v*′: 0.27, 0.54 (*xy*: 0.50, 0.44); weakly green *u*′*v*′: 0.23, 0.55 (*xy*: 0.45, 0.48); and strongly green *u*′*v*′: 0.16, 0.56 (*xy*: 0.36, 0.57).

During MEG sessions, stimuli were projected through a customized window by an InFocus IN5108 LCD back-projection system located outside the Faraday shield, onto a screen located above the participant. Participants, lying supine, viewed the screen from 113 cm. Individual “spikie” objects each had a central body of 195 pixels (5.8 degrees visual angle [dva]) wide × 175 pixels (5.2 dva) high. Their total size varied with their spikes, but the spikes never reached the border of the object image (403 × 403 pixels). Each trial’s stimulus included two “spikie” object images side by side (total size 24 × 12 dva), with a central white fixation cross (1 × 1 dva; Figure 1A). The display system was characterized in situ using a Konica Minolta CS-100A spectrophotometer and calibrated as described previously (Goddard, Mannion, McDonald, Solomon, & Clifford, 2010).

### MEG Acquisition and Eye Tracking

MEG data were collected with a whole-head MEG system (Model PQ1160R-N2, KIT) consisting of 160 coaxial first-order gradiometers with a 50-mm baseline (Uehara et al., 2003; Kado et al., 1999). Before MEG measurements, five marker coils were placed on the participant’s head. Marker positions, nasion, left and right pre-auricular points, and the participant’s head shape were recorded with a pen digitizer (Polhemus Fastrack), using a minimum of 2000 points. Each participant’s MEG data were collected in a single session of approximately 90 min, at a sampling frequency of 1000 Hz. On each trial, participants responded using a fiber optic response pad (fORP, Current Designs). We tracked participant’s eye movements using an EyeLink 1000 MEG-compatible remote eye-tracking system (SR Research, 500-Hz monocular sampling rate). Before scanning, we tested participants for their dominant eye (usually right) and focused the eye tracker on this eye.

### Experimental Protocol

Participants were instructed to covertly attend to the stimulus on either the left or right of fixation (“spatial attention” manipulation), and they were required to make a judgment based on the target object’s color or shape (“feature-selective attention” manipulation; Chen, Hoffmann, Albright, & Thiele, 2012). Each participant’s MEG session was divided into eight blocks, where the attended location and the attended feature were constant within each block. The combination of two attended locations with two attended features yielded four different tasks (Figure 1A). Before the experiment, each participant was familiarized with the object shape categories (“X-shaped” and “non-X-shaped”) and color categories (“reddish” and “greenish”) and completed a training session on a laptop outside the MEG scanner where they practiced each task.

Each trial’s stimulus included two objects, one each on the left and right of fixation, presented simultaneously. Both spatial attention (Sundberg, Mitchell, & Reynolds, 2009; Reynolds, Chelazzi, & Desimone, 1999) and feature-selective attention (Saenz et al., 2003) effects are stronger when attended and unattended stimuli simultaneously compete for access to perceptual processing. Within each block, every pairing of the 16 objects in Figure 1B was included once, giving 256 (16 × 16) trials. These 256 trials were presented in a counterbalanced order within each block, so that objects of each shape and color were equally likely to precede objects of all shapes and colors. A different counterbalanced order was used for each block, and to this sequence of 256 trials, the last trial was added to the beginning, and the first trial was added to the end, giving a total of 258 trials in each block. Data from these first and last trials were discarded.

The reported feature alternated between shape and color on every block, and the attended location alternated after the second, fourth, and sixth blocks. Starting location and feature were counterbalanced across participants. Within each pair of blocks where the attention condition was the same (e.g., Blocks 1 and 5), the buttons corresponding to the two response options were switched, so that response mappings were counter-balanced across blocks. Every block commenced with an instruction including where to attend, what feature to report, and the response mapping for that block. Before the first trial, participants were required to identify the response buttons correctly with a key press and to repeat the eye tracker’s 5-point calibration.

Every trial began with the eye tracker’s verification of fixation. Participants had to fixate within 1 dva of the fixation marker for ≥300 msec to trigger stimulus onset. During the stimulus (maximum = 150 msec) a 50 × 50 pixel white square was displayed in the bottom right (outside the stimulus region), aligned with a photodetector, attached to the mirror, whose signal was recorded with that of the gradiometers, enabling accurate alignment MEG recordings with stimulus timing. When eye-tracking registered participants were no longer fixating during the 150-msec stimulus presentation, the stimulus terminated early. Eye tracker variability (e.g., eye tracker missing frames) resulted in an unexpectedly high number of shorter trials: The median stimulus duration was 92 msec, and the first and third quartiles were 64 and 126 msec, respectively. Because this affected a majority of trials, we included all trials in our analysis, but ran an extra analysis to check that variability in stimulus duration did not account for our results (see below). After stimulus offset, the fixation marker remained white until participants responded with a button press. After the response, but no sooner than 1000 msec from the stimulus onset, the fixation marker changed for 200 msec to provide feedback: dimming to gray for “correct” or turning blue for “incorrect.” After feedback, there was a variable intertrial interval (300−800 msec), which comprised the fixation check for the subsequent trial. We used a variable intertrial interval to avoid expectancy effects.

### MEG Data Analysis: Source Reconstruction

Forward modeling and source reconstruction were performed using Brainstorm (Tadel, Baillet, Mosher, Pantazis, & Leahy, 2011; neuroimage.usc.edu/brainstorm). We created a model of each participant’s brain by manually aligning the ICBM152 template adult brain (Fonov et al., 2011) to their head shape using nasion, pre-auricular points, and head shape data. Once aligned, we applied nonlinear warping to deform the template brain to the participant’s head shape, which provides a superior model to an unwarped canonical template (Henson, Mattout, Phillips, & Friston, 2009). We generated a forward model for each model by applying a multiple spheres model (Huang, Mosher, & Leahy, 1999) to the individually warped template brain and their measured head location.

Functional data were preprocessed in Brainstorm with notch filtering (50, 100, and 150 Hz) to remove the influence of 50 Hz line noise and its harmonics, followed by bandpass filtering (0.2−200 Hz). Cardiac and eye blink artifacts were removed using signal space projection: Cardiac and eye blinks events were identified using default filters in Brainstorm, manually verified, and then used to estimate a small number of basis functions corresponding to these noise components, which were removed from the recordings (Uusitalo & Ilmoniemi, 1997). Across participants, less than 1% (0.73%) of trials contained any eye blink during the stimulus presentation, as detected from the MEG signals, and there was no evidence that they were unevenly distributed across trial types. We retained these trials to maintain the counterbalanced design for our classification analyses. From these functional data, we extracted two epochs for each trial: a measure of baseline activity (−100 to −1 msec relative to stimulus onset) and the evoked response (0−2000 msec). We estimated the noise covariance for each run from the baseline measures, regularized using the median eigenvalue, and then applied a minimum norm source reconstruction to the evoked data. For each source reconstruction, we used a 15,000 vertex cortical surface (standard for the ICBM152 template, with atlas information), dipole orientations constrained to be orthogonal to the cortical surface. We visually inspected the quality of the source reconstruction: The average trial data included an initial ERP at the occipital pole and subsequent ERPs at sources within the occipital cortex but lateral and anterior to the occipital pole, consistent with extrastriate areas along the ventral visual pathway.

### MEG Data Analysis: Preprocessing and Data Set Definitions

**Figure 2:**
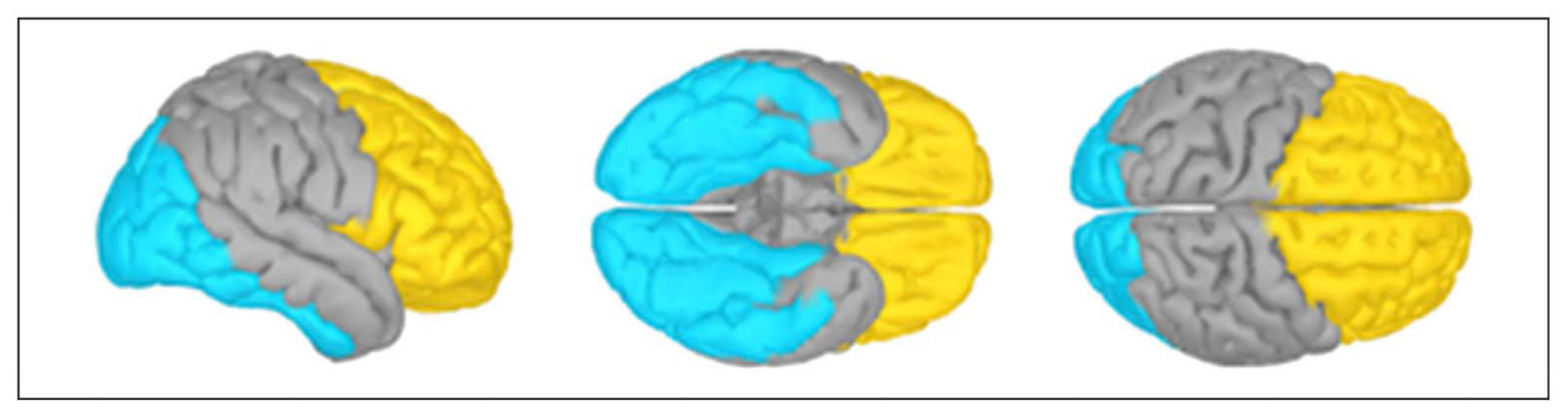
ROIs. The “occipital” (cyan) and “frontal” (yellow) ROIs shown on the partially inflated cortical surface of the ICBM152 template brain.

For classification analyses, we generated three data sets: The first included preprocessed data from all sensors, without source reconstruction; the second included sources in occipital, occipito-temporal, and inferiortemporal cortices (“occipital” ROI, 3302 vertices) in the atlas for the ICBM152 template; and the third included frontal and prefrontal cortices (“frontal” ROI, 3733 vertices), as shown in Figure 2A.

For each data set, we extracted data from −100 to 2000 msec relative to the stimulus onset of each trial. We reduced each data set, comprising 2100 msec of data for each of 2048 trials and up to 160 sensors or up to 3733 sources using PCA. We retained data from the first *n* components, which accounted for 99.99% of variance (mean = 85.3, *SD* = 6.9 for frontal ROI; mean = 76.6, *SD* = 5.8 for occipital ROI; and mean = 157.2, *SD* =1.1 for whole brain sensor data) and down-sampled to 200 Hz using the MATLAB *decimate* function.

### MEG Data Analysis: Classifier Analyses

We used classification analyses to measure the extent to which brain activity could predict task condition and the color and shape of the stimuli on each trial. For every classification, we repeated the analysis for each time sample to capture how the information carried by the neural response changed over time: We trained classifiers to discriminate between two categories of trial and tested on held-out data. We report results obtained with a linear support vector machine classifier, using the MATLAB function *fitcsvm* with KernelFunction set to linear. We also repeated our analyses with a linear discriminant analysis using the MATLAB function *classify* with type of diagLinear and obtained very similar results (not shown).

For each classification, we created “pseudotrials” by averaging across trials with the same value on the dimension of interest, but with differing values along other dimensions. We used pseudotrials to increase signal-to-noise ratio along the dimension of interest (e.g., see Guggenmos, Sterzer, & Cichy, 2018; Grootswagers, Wardle, & Carlson, 2017). When training classifiers to discriminate object color and shape, we trained and tested within a single-task condition (e.g., attend left, report color), comprising two blocks (512 trials). We trained classifiers separately on each pair of the four levels along each feature dimension, at each object location, using pseudo-trials to balance across irrelevant dimensions. For example, when classifying “strongly green” versus “weakly green” objects on the left of fixation, there were 128 “strongly green” and 128 “weakly green” trials. For classifying left object color, we defined pseudotrials that were balanced across left object shape and right object color and shape (four levels each). Because balancing across all three of these irrelevant dimensions would require 4 × 4 × 4 = 64 trials per pseudotrial, yielding only two pseudotrials per category, we instead balanced across two of three irrelevant dimensions, using 4 × 4 = 16 trials per pseudotrial, and randomized across the third (allowing eight pseudotrials per category). For each pair of irrelevant feature dimensions, we generated 100 sets of the pseudotrials, each with a different randomization. Repeating this process 3 times, balancing across different pairs of irrelevant features, gave us 300 sets of pseudotrials in total. For each of set of pseudotrials, we trained a classifier using seven of the eight pseudotrials in each condition and tested using the remaining pair of trials, repeating 8 times, averaging classifier performance across these.

For each feature dimension (color and shape), the four feature values gave six pairwise classifications, which we grouped according to the feature difference between the pair. When considering the effects of spatial and feature-selective attention across feature difference, we grouped classification pairs according to whether they were one (three pairs), two (two pairs), or three (one pair) steps apart along their feature dimension and averaged across classifications within each group.

To summarize the effects of spatial attention (***SpatAtt***) and feature-selective attention (***FeatAtt***), we used the following metrics, based on classifier performance (*d*′) in the attended location, attended feature (*aLaF*) condition; the attended location, unattended feature (*aLuF*) condition; the unattended location, attended feature (*uLaF*) condition; and the unattended location, unattended feature (*uLuF*) condition.

(1) SpatAt=aLaF+aLuF−uLaF−uLuF

(2) FeatAt=aLaF+uLaF-aLuF-uLuF

### Statistical Testing

To generate a null distribution of chance classifier performance for statistical testing, we repeated analyses within each participant’s data using randomly permuted trial labels (10 permutations of the data from every fourth time sample, a total of 1060 samples of permuted data per participant). For each of the 1060 permuted data sets, we then averaged classification accuracy across participants to generate a group-level null distribution of 1060 values. We visualized the null distribution over time and found no indication that permuted data from different time samples varied in chance performance, so we collapsed these across time in statistical testing, rather than repeating for each time sample, to reduce the time taken for these intensive computations. Across classifications, average chance performance varied from *d*′ = 0.000 to a maximum of *d*′ = 0.015. We used this group-level null distribution of 1060 values to calculate one-sided nonparametric *p* values for the probability that observed group mean classifier performance occurred by chance and to generate a null distribution of 1060 F statistics against which to compare the observed *F* statistics from repeated-measures ANOVAs: the main effects of Attended Location and Attended Feature on classifier performance, and the interaction between these main effects: *F*(1,19) in each case. Similarly, we also calculated *SpatAtt* and *FeatAtt* using the classifier performance for data with permuted trial labels and used these to generate null distributions of *F* statistics for the interaction between feature difference and attention type. When using repeated-measures ANOVAs to test for main and interaction effects (Figures 4 and 5), we report nonparametric *p* values based on the proportion of *F* statistics in the null distribution that exceeded the observed value. In each case, we corrected these *p* values for multiple comparisons across time samples using a false discovery rate (FDR) correction (Benjamini & Hochberg, 1995).

#### Control Analysis: Effect of Variable Stimulus Durations

Stimuli were presented for variable durations across trials, because trials were terminated when the participant broke fixation or the eye tracker dropped a frame. To check that the extent to which variability could potentially drive the classification results reported, we repeated each classification analysis above using the stimulus state (on or off) for each time sample of each trial, instead of the neural data. For each participant, we constructed an alternate data set, where each trial’s data were a single, binary dimension (0 and 1 according to whether the stimulus was on or off at each time sample). Using this alternate data, we ran all classifications following identical procedures, including pseudotrials, following a “same analysis approach” (Görgen, Hebart, Allefeld, & Haynes, 2018).

### Modeling the Effects of Spatial and Feature-selective Attention on Population Representations of Shape and Color

**Figure 3:**
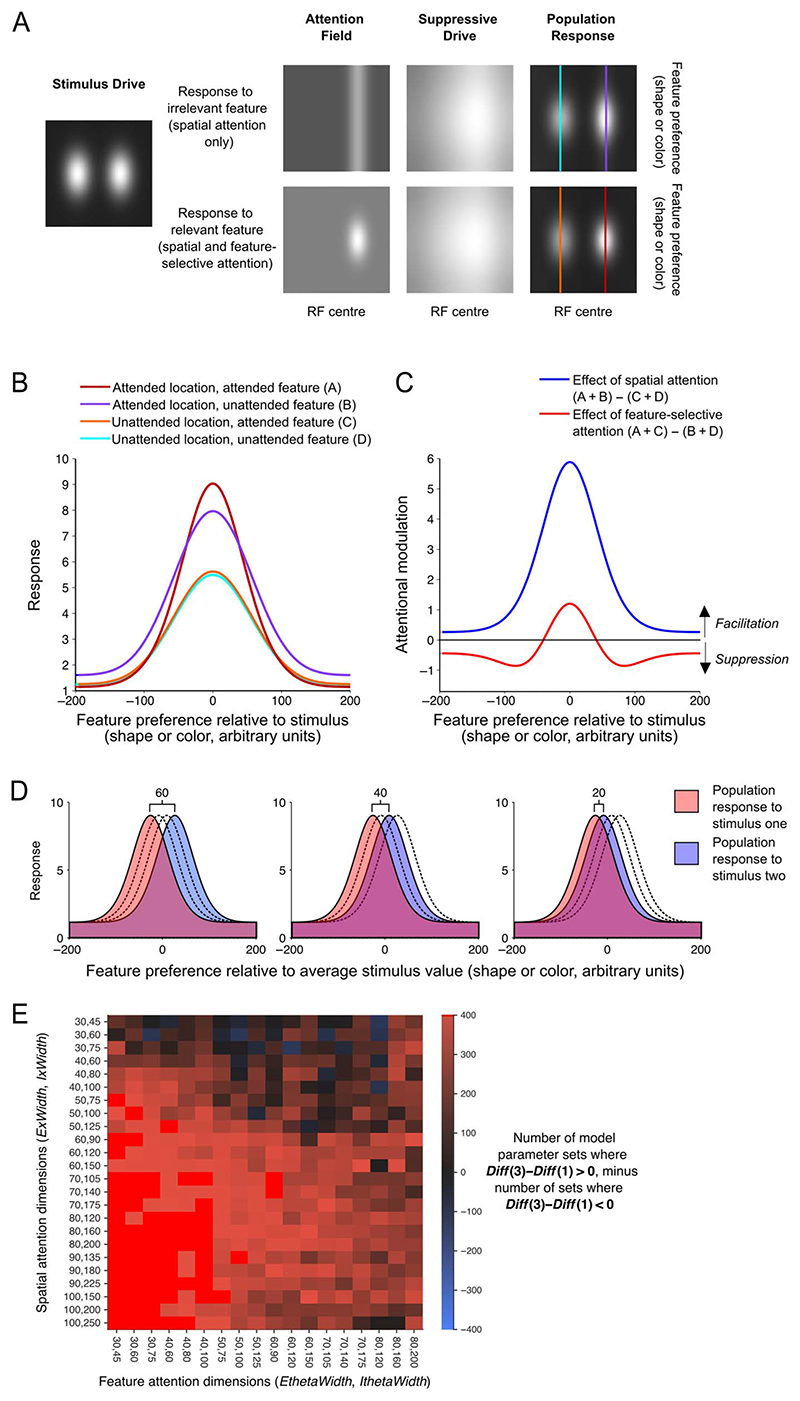
Normalization model of attention. (A) Illustration of each of the model elements from Reynolds and Heeger (2009, Figure 1), for a set of example model parameters, where each grayscale image depicts a matrix of values varying along a spatial dimension (horizontally) and a feature dimension (vertically). For each set of model parameters, we generated a single “stimulus drive” and two versions of the “attention field,” which lead to subtly different “suppressive drives” and “population responses.” From these two population responses, we derived curves predicting the population response as a function of each neuron’s preferred feature value for each of the four attention conditions (the columns of the matrix indicated with different colored vertical lines in A). These population responses are replotted as line plots in B. In (C), the predicted effects of spatial and featurebased attention on the population response are summarized as the difference between relevant population curves from B. (D) We predicted classifier performance in each attention condition by centering the population response from B on four different stimulus feature values and predicting classifier performance when discriminating between population responses to stimuli of that were 60, 40, or 20 (arbitrary) units apart along the feature dimension to simulate the population response to stimuli that were three, two, or one step apart in either color or shape. We predicted classifier performance (d^0^) using the separation of the two population responses, in a manner analogous to that used in signal detection theory. (E) The model predictions across four model parameters: the excitation and inhibition width of the spatial and featurebased attention fields (*ExWidth*, *IxWidth*, *EthetaWidth*, and *IthetaWidth* in Table 1). In each cell, there were 400 sets of model parameters (where other model parameters were varied). For each set of model parameters, we calculated the difference between attention effects (*Diff* = *SpatAtt* − *FeatAtt*) across feature difference (as in Figure 4). Here, we show a number of model parameter sets for which the pattern of results was qualitatively similar to the average model prediction (Figure 4B) and to the data (e.g., Figure 4E). That is, model sets where *Diff* at three steps (*Diff*(3)) minus *Diff* at one step difference (*Diff*(1)) was positive (red cells, 95% of cases). There were also some combinations of excitation and inhibition widths for which all 400 cases followed this pattern (bright red cells, 16% of cases).

We examined whether a normalization model of the effects of attention at the cellular level could capture the differences we observed across subtypes in how attention affected stimulus-related information in the population response. In nonhuman primates, spatial attention’s effect on the tuning of individual neurons has been characterized as multiplicative response gain (Lee & Maunsell, 2010; McAdams & Maunsell, 1999; Treue & Martinez-Trujillo, 1999), contrast gain (Martinez-Trujillo & Treue, 2002; Reynolds, Pasternak, & Desimone, 2000), or both (Williford & Maunsell, 2006). The effects of spatial attention on contrast response functions measured with fMRI are also mixed (Li, Lu, Tjan, Dosher, & Chu, 2008; Buracas & Boynton, 2007). A recent EEG study reported evidence that covert spatial attention induces spatially selective response gain in the population response (Foster et al., 2021). In contrast, feature-based attention produces single-unit effects, which should produce a “sharpening” of the population response around the attended feature value (Martinez-Trujillo & Treue, 2004), as was recently reported with MEG (Bartsch et al., 2017). Intuitively, we expected that these effects might be consistent with the different patterns of enhancement we observed in the present data. To formalize this intuition and to test whether these single-unit effects could manifest in the patterns of difference we observed, we implemented the Reynolds and Heeger (2009) normalization model of attention to generate predictions for our design, as illustrated in Figure 3.

We started with the MATLAB routines from Reynolds and Heeger (2009) available from www.cns.nyu.edu/heegerlab/. Because we did not have strong a priori predictions for many of the model parameters, we tested a broad range of plausible model parameters (see Table 1). For each set of model parameters (172,800 sets in total), we used the model to predict the response of the neural population as a function of stimulus feature preference (along the shape or color dimension), for each of four cases, illustrated by lines of different colors in Figure 3A, B. In every case, the stimulus was a single feature value (a specific color or shape) at two fixed locations (left and right of fixation). In two cases, we simulated attention to one location in the absence of any feature-based attention (simulating attention to the orthogonal feature dimension). In the other two cases, we simulated attention to one location and attention to the feature value of the stimuli. From these, we predicted the population response at attended and unattended locations, in the presence and absence of feature-based attention. As illustrated in Figure 3C, according to the model, spatial attention tends to boost the population response as a multiplicative scaling of the original response, whereas feature-based attention produces both facilitation and suppression of the response, which leads to sharpening of the population response around the attended value. Note that in the model the spatial and feature dimensions are affected by attention in equivalent ways, with within-dimension attention leading to a sharpening of the population response along the attended dimension. In this way, spatial attention can also lead to a sharpening of the population response along the spatial dimension, but only feature-based attention leads to sharpening along the feature dimension.

One difference between the model (Reynolds & Heeger, 2009) and our experiment is that the model is designed to capture feature-based attention (attending to a specific feature value, e.g., red), whereas we manipulated feature-selective attention (attending to a feature dimension, e.g., color). Although feature-based attention has received greater attention in the electrophysiology literature, feature-selective attention has been demonstrated to have similar effects at the level of single neurons (Cohen & Maunsell, 2011) and to produce changes in human EEG (Verghese, Kim, & Wade, 2012) and fMRI (Scolari, Byers, & Serences, 2012) responses that are similar to the effects of feature-based attention. Furthermore, although in human studies feature-based attention usually refers to attention to a particular feature value that is known before stimulus onset (e.g., visual search for red objects), in the electrophysiological literature, there are seminal works (e.g., Martinez-Trujillo & Treue, 2004) that explored the effects of feature-based attention by manipulating the feature value of an attended stimulus and testing the effects on responses to unattended stimuli of the same or different feature value. In our results (below), the effects of feature-selective attention emerged after the initial stimulus-induced response, making it more likely that the observed effects were occurring after participants had engaged their attention with the specific feature value of the stimulus. In these ways, we felt it appropriate to see whether the effects of feature-selective attention we observed could be captured by modeling the effects of attending to the feature value of the stimulus. We therefore implemented the feature-selective attention manipulation in the model by generating population responses to two stimuli of the same feature value and modeling the presence of feature-selective attention as feature-based attention to that feature value.

For every predicted population response, we predicted classifier performance when discriminating responses to stimuli of different feature values. To do this, we compared two population responses that were identical, except that they were centered on different feature values, as shown in Figure 3D. To simulate the three steps of stimulus difference, we considered cases where the centers of the population responses were separated by 20, 40, or 60 in the arbitrary units of the feature dimension. In the case of stimuli varying in color, the chromaticity coordinates of the stimuli varied from strongly red *u*′*v*′ : 0.35, 0.53, to strongly green *u*′*v*′: 0.16, 0.56, which means that, for the model, we were treating a difference of 60 arbitrary units as a distance of approximately 0.19 in the *uv* chromaticity plane. For shape, the feature dimension is defined by the transition from “X-shaped” to “non-X-shaped.” We are not asserting that there exist neurons tuned to this novel complex shape dimension in the same way as there are neurons tuned to color, but for the purposes of the model, we treated these dimensions as equivalent. Because participant performance was similar for the color and shape task, we used the same distances (20, 40, and 60 in the arbitrary units) to avoid adding another parameter to the modeling results.

Using the pairs of population responses (such as those in Figure 3D), we predicted classifier performance (*d*′) using the separation of the two population responses, in a manner analogous to that used in signal detection theory. To determine *d*′ for these population responses, we calculated a “hit rate” for an optimal observer detecting a signal (stimulus two) among noise (stimulus one), where their criterion (*c*) is at the midpoint between the peaks of the two curves. We defined the “hit rate” *(hits)* as the area under the blue curve to the right of *c* and the “false alarm rate” (FA) as the area under the red curve to the right of *c*. Then the predicted classifier performance *d*′ = norminv(hits) − norminv(FA). In this way, for each set of model parameters, we predicted classifier performance in each attention condition, for each of the three step sizes in feature difference.

From the predicted classification performance, we summarized the predicted effects of spatial attention and feature-selective attention using the ***SpatAtt*** and ***FeatAtt*** values from Equations 1 and 2. Across these different parameter sets, there was variation in the predicted magnitude of the effects of spatial attention and feature-selective attention, and there was also variation in which stimulus pair feature distances (step sizes) showed the greatest enhancement. However, when compared with spatial attention, feature-selective attention tended to produce relatively more enhancement of small stimulus feature differences than larger ones, as seen in the average difference across all model parameter sets (Figure 5E). As seen in Figure 3E, a majority of model parameter sets (83%) showed this qualitative pattern of relative enhancement across attention subtypes.

### MEG Data Analysis: Granger Analysis of Feedforward and Feedback Information Flows

We tested for temporal dependence between the patterns of classifier performance in occipital and frontal data sets, seeking evidence of information flows from occipital to frontal cortices (feedforward) and from frontal to occipital cortices (feedback), following the rationale developed in earlier work (Karimi-Rouzbahani, 2018; Goddard, Carlson, Dermody, & Woolgar, 2016). Specifically, we tested for Granger causal relationships between the patterns of classifier performance based on the occipital and frontal data sets. We summarized the color and shape information for each region (occipital and frontal), for each time sample, as a 6 × 4 dissimilarity matrix (DSM) of classifier performances. For both color and shape, the 6 × 4 DSM was defined as each pairwise comparison (six classifications across the four levels of the feature) by four attention conditions (*aLaF*, *aLuF*, *uLaF*, *uLuF*).

The logic of Granger causality is that time series X “Granger causes” time series Y if X contains information that helps predict the future of Y better than information in the past of Y alone (for a recent review of its application in neuroscience, see Friston, Moran, & Seth, 2013). We performed a sliding window analysis of a simplified (special case) of Granger causality using the partial correlations in Equations 3 and 4 to define feedforward (***FF***) and feedback; (***FB***) information flows for each time sample (***t***).

(3) $$FF(t,d,w)=\text{ρ}DSM_{\left(\text{frontal},t\right)}DSM_{\left(\text{occipital},t,d,w\right)}DSM_{\left(\text{frontal},t,d,w\right)}$$

(4) $$FB_{\left(t,d,w\right)}=\text{ρ}DSM_{\left(\text{occipital},t\right)}DSM_{\left(\text{frontal},t,d,w\right)}DSM_{\left(\text{occipital},t,d,w\right)}$$

where *DSMy*_(***loc, t***)_ is the *DSM* based on the sources at location ***loc*** at time ***t*** msec post stimulus onset, and *DSM*_(***loc, t, d, w***)_ is the DSM based on the sensors at location ***loc***, averaged across all time samples from ***t*** msec to ***t*** − (***d*** + ***w***) msec post stimulus onset. We calculated ***FF*** and ***FB*** for 30 overlapping windows: for five window widths (***w*** = 10, 20, 30, 40, or 50 msec) for each of six delays *(**d*** = 50, 60, 70, 80, 90, or 100). We tried a range of values for ***w*** and ***d*** to capture interactions between occipital and frontal cortices that may occur at different timescales. Because the results were broadly similar across values of ***w*** and ***d***, we report ***FF*** and ***FB*** values averaged across all values of ***w*** and ***d***.

We report the results of this analysis in terms of the difference between the feedforward and feedback information flows (*FF* − *FB*). To assess whether this difference was significantly above or below chance, we generated a null distribution of this difference for every time sample by performing the same analysis on 1000 bootstraps of data from each participant where the exemplar labels were randomly permuted for each of the DSMs used in Equations 3 and 4.

## Results

We acquired MEG recordings while participants categorized the color (reddish or greenish) or shape (X-shaped or non-X-shaped) of a series of stimuli that were either closer or farther from these decision boundaries.

### Behavioral Accuracy and RT

Participants were faster and more accurate at identifying color and shape for objects that were far from the decision boundary relative to those that were near the decision boundary. For the color task, the average accuracy was 95.6% (*SD* = 3.6%) on the easy trials and 85.2% (*SD* = 7.3%) on the hard trials. Similarly, for the shape task, the average accuracy was 94.1% (SD = 3.5%) on the easy trials and 74.1% (SD = 4.7%) on the hard trials. A three-way repeated-measures ANOVA of accuracy across Task (color or shape), Difficulty (easy or hard), and Attended Object Location (left or right) showed significant main effects of Task, *F*(1, 19) = 41.1, *p* <.001, and Difficulty, *F*(1, 19) = 328.4, *p* <.001, but not Location, *F*(1, 19) = 1.1, *p* =.32, and a significant interaction between Task and Difficulty, *F*(1, 19) = 47.4, *p* <.001. Follow-up simple main effects showed there was a significant effect of Difficulty on accuracy for both the color task, *F*(1,19) = 67.4, *p* <.001, and the shape task, *F*(1, 19) = 525.1, *p* <.001.

RTs were also modulated by task difficulty. For the color task, median RT was 0.69 sec on the easy trials and 0.81 sec on the hard trials, and for the shape task, the median RT was 0.74 sec and 0.82 sec on the easy and hard trials, respectively. We performed a three-way repeated-measures ANOVA of the effects of Task, Difficulty and Attended Location on log RT. Again, there were significant main effects of Task, *F*(1, 19) = 7.0, *p* =.016, and Difficulty, *F*(1, 19) = 171.7, *p* <.001, but not Location, *F*(1, 19) = 1.2, *p* =.28, and a significant interaction between Task and Difficulty, *F*(1, 19) = 16.0, *p* <.001. Follow-up simple main effects showed there was a significant effect of Difficulty on RT for both the color task, *F*(1,19) = 184.5, *p* <.001, and the shape task, *F*(1,19) = 56.9,*p* <.001. On 77% of trials, the RT was shorter than 1 sec, and the feedback onset was 1 sec.

### Classification Analyses of MEG Data

We trained classifiers to make a series of orthogonal discriminations to quantify neural information about the participant’s task and the stimulus, within the two ROIs. We could robustly decode the participant’s task from both occipital and frontal sources, indicating that neural responses differed according to the attentional set of the participant. Decoding of attended location (left vs. right) peaked at 270 and 390 msec after stimulus onset (occipital and frontal ROIs, respectively) and a decoding of attended feature (color vs. shape) peaked at 455 msec after stimulus onset in both ROIs. Below, we present the effects of the attentional manipulations on the representation of object color and shape.

### Spatial and Feature-selective Attention Interact Multiplicatively to Boost Information Processing

**Figure 4:**
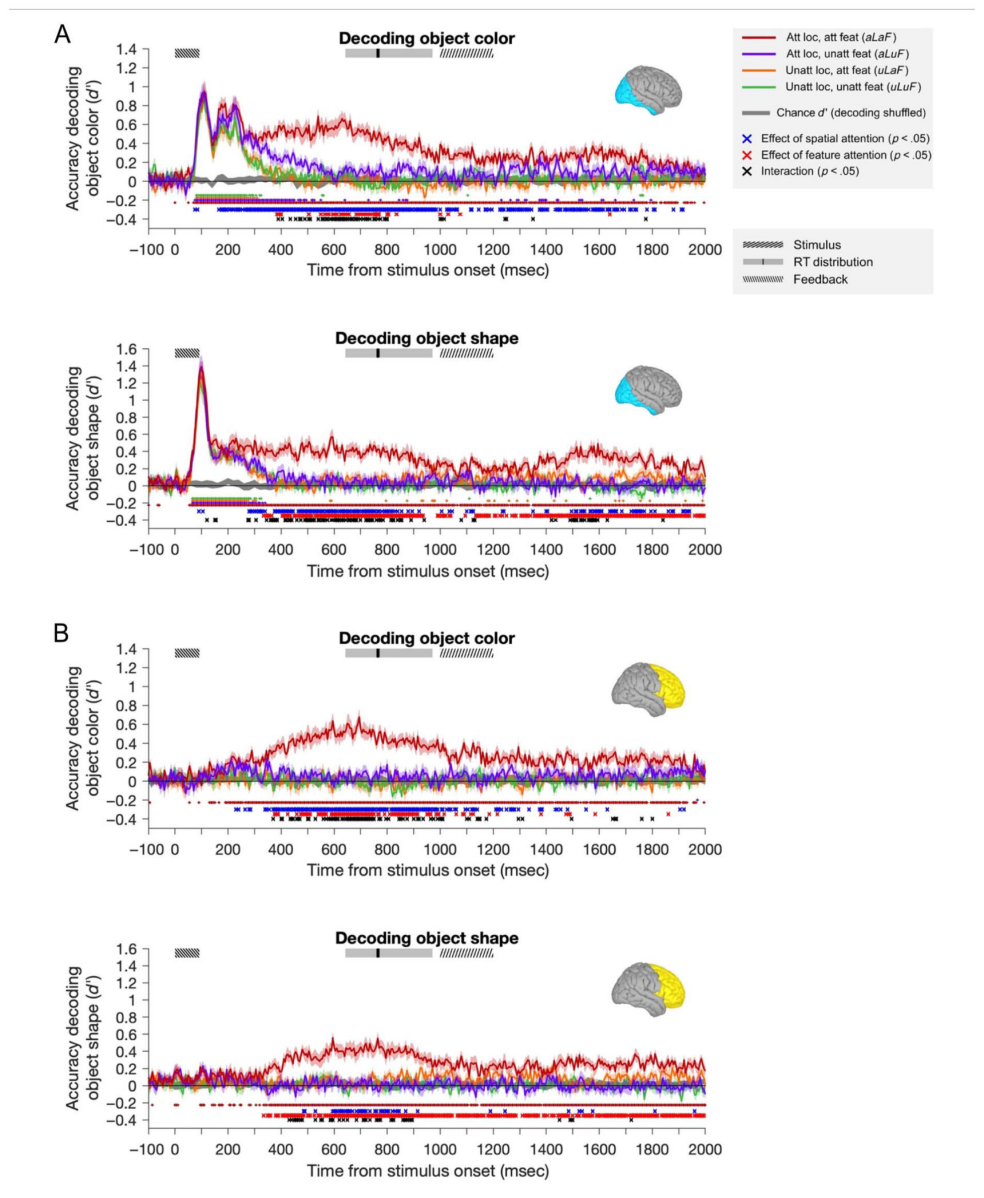
Classifier performance across participants (*n* = 20) for decoding object features. For both occipital (A) and frontal (B) ROIs, classifiers were trained to discriminate the color (top plots) and shape (bottom plots) of attended and unattended objects. Classifier performance is shown for each attention condition separately: attended location, attended feature (*aLaF*); attended location, unattended feature (*aLuF*); unattended location, attended feature (*uLaF*); and unattended location, unattended feature (*uLuF*). Shaded error bars indicate the 95% confidence intervals (between-subject mean). At the top of each plot, boxes indicate the time of the stimulus presentation (shaded area indicates onset until the median duration of 92 msec), the RT distribution (shaded area includes RTs within the first and third quartiles, black line indicates median RT), and the time during which participants received feedback on their accuracy on those trials where their RT was <1 sec (77% of trials). On trials where RT was >1 sec (23% of trials), the 200-msec feedback started at the time of response. The shaded gray region around the *x*-axis indicates the 95% confidence intervals of the four classifications when performed on randomly permuted data (the empirical null distribution). Small dots below each plot indicate time samples for which the classification of matching color was above chance level (FDR corrected, *q* <.05). Below these, crosses indicate time samples for which there was a significant effect (FDR corrected, *q* <.05) of spatial attention (blue asterisks), feature attention (red asterisks), or an interaction of the two (black asterisks).

First, we examined the dynamics with which spatial and feature-selective attention affected object information processing and how the two subtypes of attention interacted in affecting this neural signal. To do so, we trained classifiers to discriminate the color and shape of the attended and nonattended objects. Figure 4 shows the representational dynamics of object color and shape information, for each of the four attentional conditions (2 spatial locations × 2 tasks) in each ROI.

For both object color and object shape, we found significant main effects of Spatial Attention and Feature Attention and significant interactions between these effects (at times shown in Figure 4: blue, red, and black crosses, respectively, based on repeated-measures ANOVAs compared with a permutation-based null distribution, see Methods for details). In the occipital ROI, spatial attention produced a small but significant increase early in the decoding of both color and shape (blue crosses <100msec in Figure 4A, at 75 and 85 msec for decoding color, and 90 and 105 msec for decoding shape) at or just before the earliest peak in information processing (which was at 105−110 msec for color and 95−100 msec for shape). There was no corresponding increase attributable to feature-selective attention. For color coding, there was also a secondary early peak in coding (~ 165−240 msec), at which time there were again significant effects of Spatial, but not Feature, Attention. Coding in the frontal lobe was not above chance (and not modulated by attention) at these early time points.

For both stimulus features and ROIs, the attention effects of greatest magnitude emerged later, from ~300 msec after stimulus onset. In the occipital lobe, from this time point on, the representation of task-relevant stimulus-related information (Figure 4A, red traces) was sustained, whereas the equivalent information in all other attentional conditions was attenuated. In the frontal ROI, at this time, there emerged a selective representation of information about the attended feature at the attended location (Figure 4B, red traces). In the occipital ROI, the sustained effects of spatial attention preceded those of feature-selective attention for both color (spatial from 165 msec, feature from 385 msec) and shape (spatial from 280 msec, feature from 335 msec).

From around 400 msec, for both occipital and frontal regions (slightly earlier for shape in the occipital ROI), there was a significant interaction between the effects of spatial and feature-selective attention, which indicated that the two effects combined in a multiplicative rather than an additive manner (black crosses). In general, whenever both spatial and feature-selective attention had significant effects, there was also an interaction. In both cases (color and shape), the sustained effects of spatial and feature-selective attention interacted multiplicatively to selectively boost in the decoding of the attended feature at the attended location, with little enhancement in classifier performance when only feature or location was selected. That is, when location but not feature was attended (purple lines) or when feature but not location was attended (orange lines), decoding was closer to the completely unattended condition (green lines) than to the fully attended condition (dark red lines).

Information about each attended feature at the attended location (dark red lines in Figure 4) also had later peaks in both the occipital ROI (540−630 msec) and the frontal ROI (595−695 msec). These peaks are well after the offset of the stimulus (92 msec) and just before the median RT (770 msec), suggesting they may be associated with the participant’s decision and/or the remembered feature value. We balanced the response mapping (by switching the keys associated with each response pair on half the runs and creating pseudotrials, which averaged across equal numbers of trials from each response mapping), meaning that the motor preparation associated with the participants’ response cannot have contributed to this effect.

In summary, at early time points, all visual information (shape and color of both objects) was represented in the MEG trace, with some evidence for a weak modulation of this information by spatial attention alone. At later times, both spatial and feature-selective attention had robust effects on coding of both shape and color in both ROIs. These effects were multiplicative rather than additive, leading to a selective representation of the attended feature of the attended object, which was sustained for much of the epoch.

### Control Analysis: Variable Stimulus Durations Cannot Explain Observed Classifier Performance

Because trials were terminated when the participant broke fixation or the eye tracker dropped a frame, stimuli were presented for variable durations across trials. To check that this variability could not drive the classification results reported above, we first plotted the average duration for each stimulus and condition and checked that there were no identifiable differences between conditions (not shown). Then, as a stronger test, we repeated each classification analysis above using the stimulus state (on or off) for each time sample of each trial, instead of the neural data (see Methods). Across time samples and classifications, the maximum group-average classifier sensitivity was *d*′ = 0.4, indicating that variability in stimulus duration could have made a small contribution to overall classifier performance. However, there was very little difference between classifier accuracy for different attention conditions or across step sizes. When we performed the statistical tests reported in Figure 4 on the stimulus duration data, the only significant result (effect of attended location for decoding stimulus color) was in the opposite direction (decoding was higher for unattended than attended locations).

### Spatial and Feature-selective Attention Have Distinct Effects on Population Tuning Profiles

**Figure 5:**
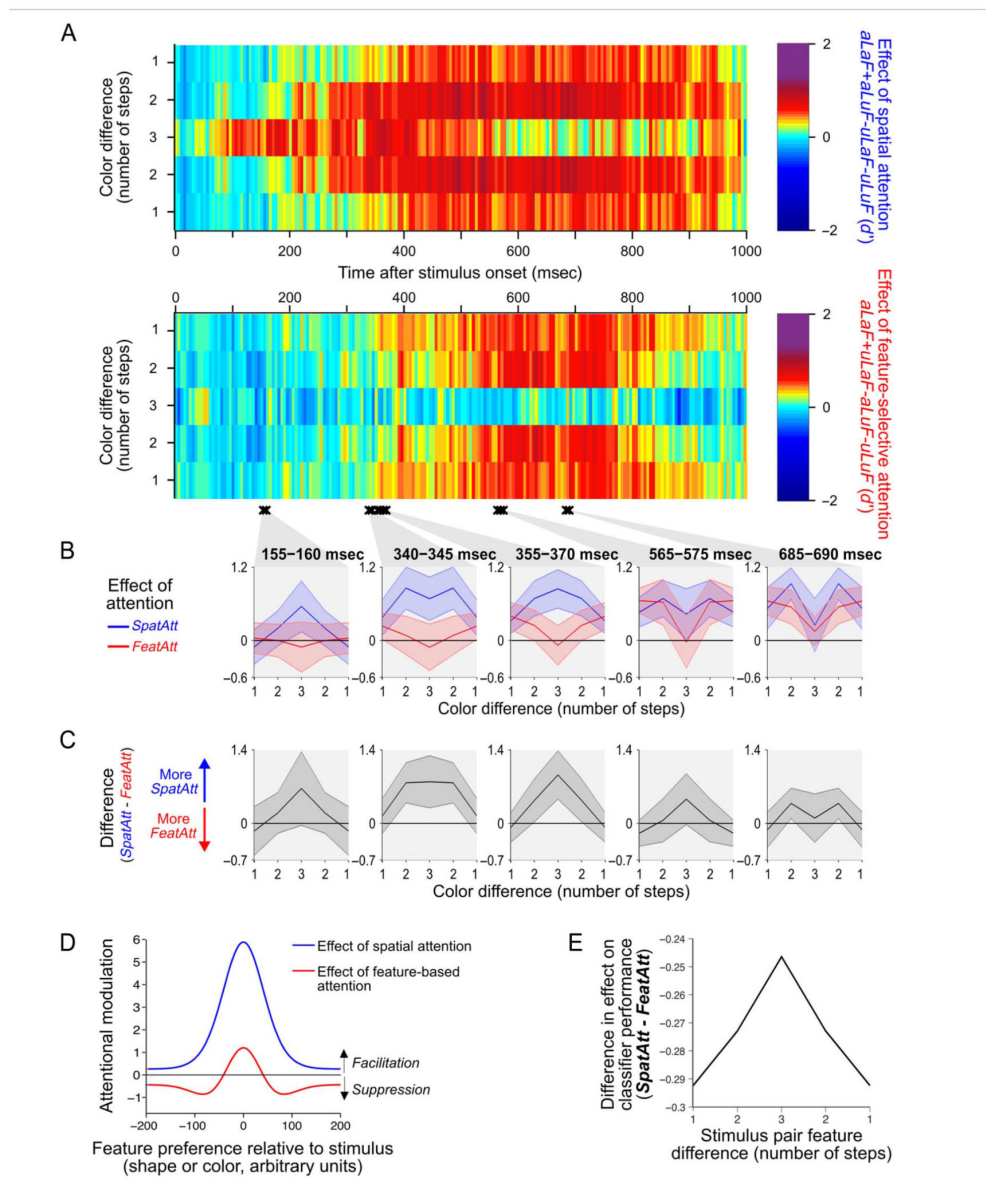
Effects of spatial and feature-selective attention on the decoding of object color in the occipital ROI. (A) The effects of spatial attention (top plot) and feature-selective attention (bottom plot) on decoding of stimulus color were calculated by taking the difference in classifier performance (*d*′) between the relevant attended and unattended conditions for each step size (see Equations 1 and 2). Two-way repeated-measures ANOVAs for each time sample revealed times where there was a significant interaction (compared with a permutation-based null distribution) between Attention Condition and Step Size (black crosses show clusters of at least two time samples where *p* <.05). Data from four epochs of interest, with significant interactions, were averaged and plotted in the insets below B. In C, the difference between the two attention effects (from the same time bins as in B) is plotted. Data in A−C are mirror-reversed for illustration only; statistical analyses were performed on data without mirror reversals. Shaded error bars indicate the 95% confidence interval of the between-subject mean. (D) The predicted change in simulated population response induced by spatial and feature-based attention on a population of neuronal responses, for an example set of normalization model parameters. According to the model, spatial attention tends to boost the response of all neurons as a multiplicative scaling of the original response, whereas feature-based attention produces both facilitation of neurons, which prefer the attended value, and suppression of neurons preferring nearby values, which leads to sharpening of the population response around the attended value. (E) Predicted difference between the effects of spatial (*SpatAtt*, Equation 1) and feature-selective attention (*FeatAtt*, Equation 2) on classifier performance across pairs of stimuli with different physical differences, averaged over all 172,800 sets of model parameters we tested. The difference values plotted in C correspond to the prediction from the model in E.

Next, we considered whether spatial and feature-selective attention differ in the way they shape how stimulus information in represented in population codes in human cortex. To explore this, we considered how classifier performance varied with the physical difference in the stimuli being discriminated. Because our stimuli varied in four “steps” along both color and shape dimensions, the pairs of object stimuli that classifiers were trained to discriminate could be one, two, or three steps apart along either dimension. Classifier performance generally increased with physical difference (data not shown). Additionally, we found that the effects of spatial and feature-selective attention varied according to the physical discriminability of the stimuli (Figure 5).

Figure 5A shows the effect of each type of attention separately, across step size and time, for coding of object color in the occipital ROI. The effects of attention are here expressed as the change in classifier performance between attended and unattended conditions, so a difference of zero (light green in Figure 5A) shows no difference between attention conditions, rather than an overall classifier performance of zero. If spatial and feature-selective attention produced similar effects on neural responses, then the two plots in Figure 5A should look similar, and the regions of yellow−red (largest improvements in decoding with attention) should have a similar shape. Instead, there are systematic differences between the two in their relative effects on classifier performance across step size. This is seen most clearly in the “convex” versus “concave” shape of the yellow−red regions from 300 msec after stimulus onset. Although spatial attention tended to produce greatest enhancements for stimuli separated by two steps in feature space, feature-selective attention tended to produce greatest enhancements for stimuli only one step apart.

To identify times at which spatial and feature-selective attention differed statistically in their effects across step size, we performed a two-way repeated-measures ANOVA compared with a permutation-based null distribution for each time sample (see Figure 5A, black crosses, for times of significant interaction between Attention Type and Step Size). Then, for each cluster of time samples with significant interactions, we plotted the average effects of spatial and feature-selective attention (Figure 5B). We found that the effect went in the same direction for every cluster: spatial attention had a greater effect than feature-selective attention at the largest step size (size 3), whereas feature-selective attention had a larger effect than spatial attention at the smallest step size (size 1). This is illustrated most clearly in the difference plots of Figure 5C. As an additional control, we confirmed that the same pattern of results persisted when excluding participants with any bias to fixate toward the attended location (data not shown). These data suggest a robust difference between spatial and feature-selective attention in the way they enhance the color information in occipital areas.

**Figure 6:**
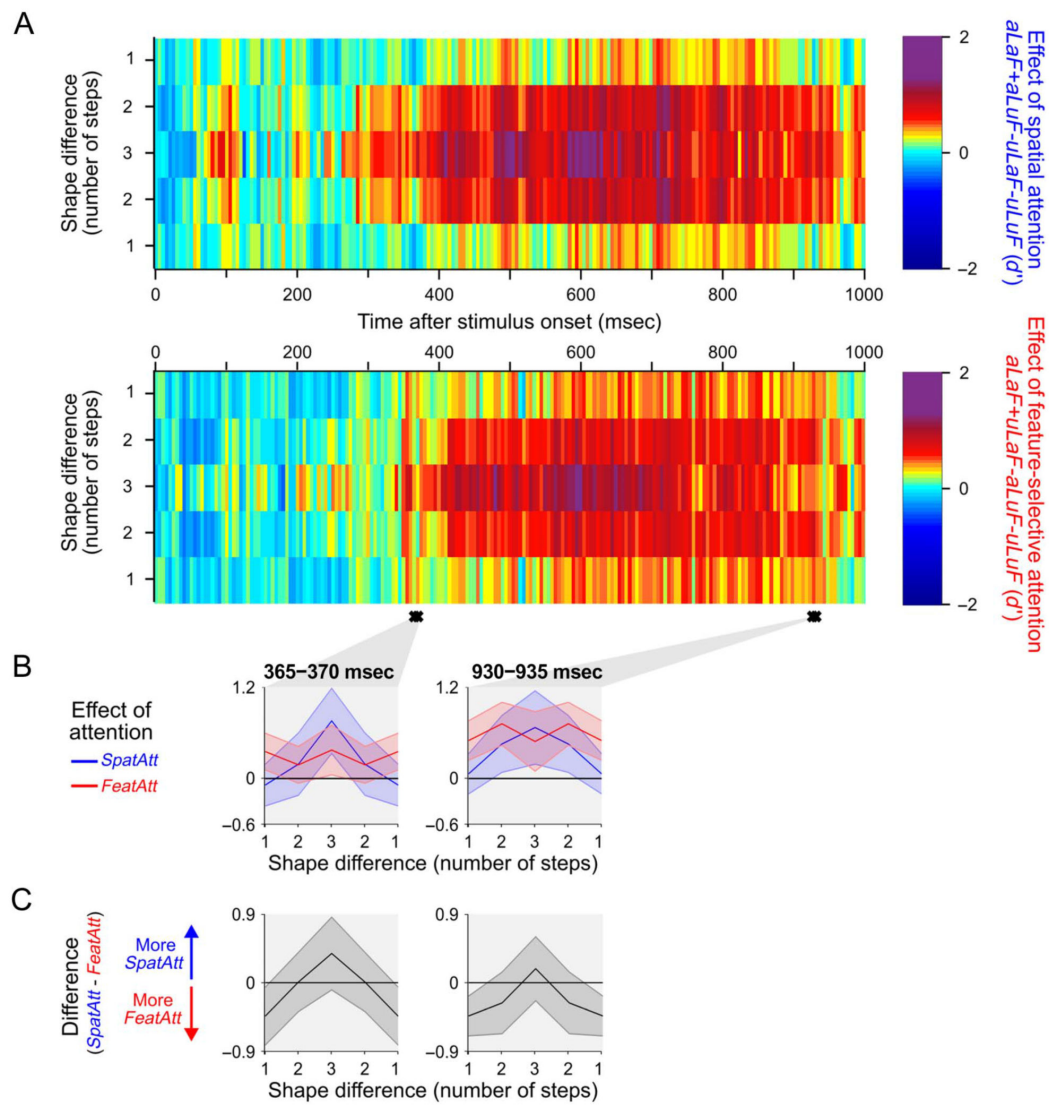
Effects of spatial and feature-selective attention across decoding of object shape for all MEG sensors. Plotting conventions for A−C are as in Figure 5A-C.

Next, we asked whether the same pattern of effects was seen for coding of shape information. In both the occipital and frontal ROIs, the effects of spatial and feature-selective attention were more uniform across step sizes, and there were no clusters of time samples with a significant interaction between attention subtype and step size (data not shown). In a more powerful analysis pooling over data from the whole brain (sensor level; see Methods), there were two clusters of consecutive time samples where there was a significant interaction between attention subtype and step size (Figure 6). Overall, any pattern of difference was much weaker for shape than for color (see Figure 5A vs. Figure 6A). However, where these interactions occurred, the pattern of effects was in the same direction as that shown in Figure 5C. The data, though less definitive than for color, offer preliminary support for the notion that there may be a general difference between spatial and feature-selective attention in their effect on population-level tuning.

We were interested to know whether this distinction between the effects of spatial and feature-selective attention at the population level might reflect differences between spatial and feature attention in their effects on the tuning of individual neurons. To test this idea, we used a normalization model of attention (Reynolds & Heeger, 2009; see Methods for details). A number of groups have proposed models including normalization to describe the effects of attention on neuronal response properties (Boynton, 2009; Lee & Maunsell, 2009; Reynolds & Heeger, 2009).

Model predictions for our experimental design are illustrated in Figure 5D-E. Details of the model predictions, including further illustrations, are found in Figure 3. Because the model of Reynolds and Heeger (2009) is descriptive, with a large number of free parameters, we systematically generated model predictions for a wide range of model parameter sets, 172,800 in total. The large space of model parameter sets generally converged on the prediction that when compared with spatial attention, feature-selective attention would produce relatively more enhancement of small physical stimulus differences than larger ones (Figure 5E), matching the pattern of difference across attention types in our data.

### Frontal Activity Influences the Occipital Representation of Object Shape and Color with a Time Course Matching the Strongest Attentional Effects

To characterize the exchange of stimulus-related information between the occipital and frontal ROIs, we used an information flow analysis (Goddard et al., 2016). Because we have fine temporal resolution measures of each pairwise classification, in each attention condition, we used the pattern of classification performance across these measures as a summary of the structure of representational space for each time sample and tested for evidence of Granger causal interactions between the ROIs (see Methods for details). Note that by applying this analysis to patterns of classification performance (rather than raw signals), we are not simply testing for evidence of connectivity between brain regions but are specifically testing for evidence of the exchange of stimulus-related information between areas.

**Figure 7:**
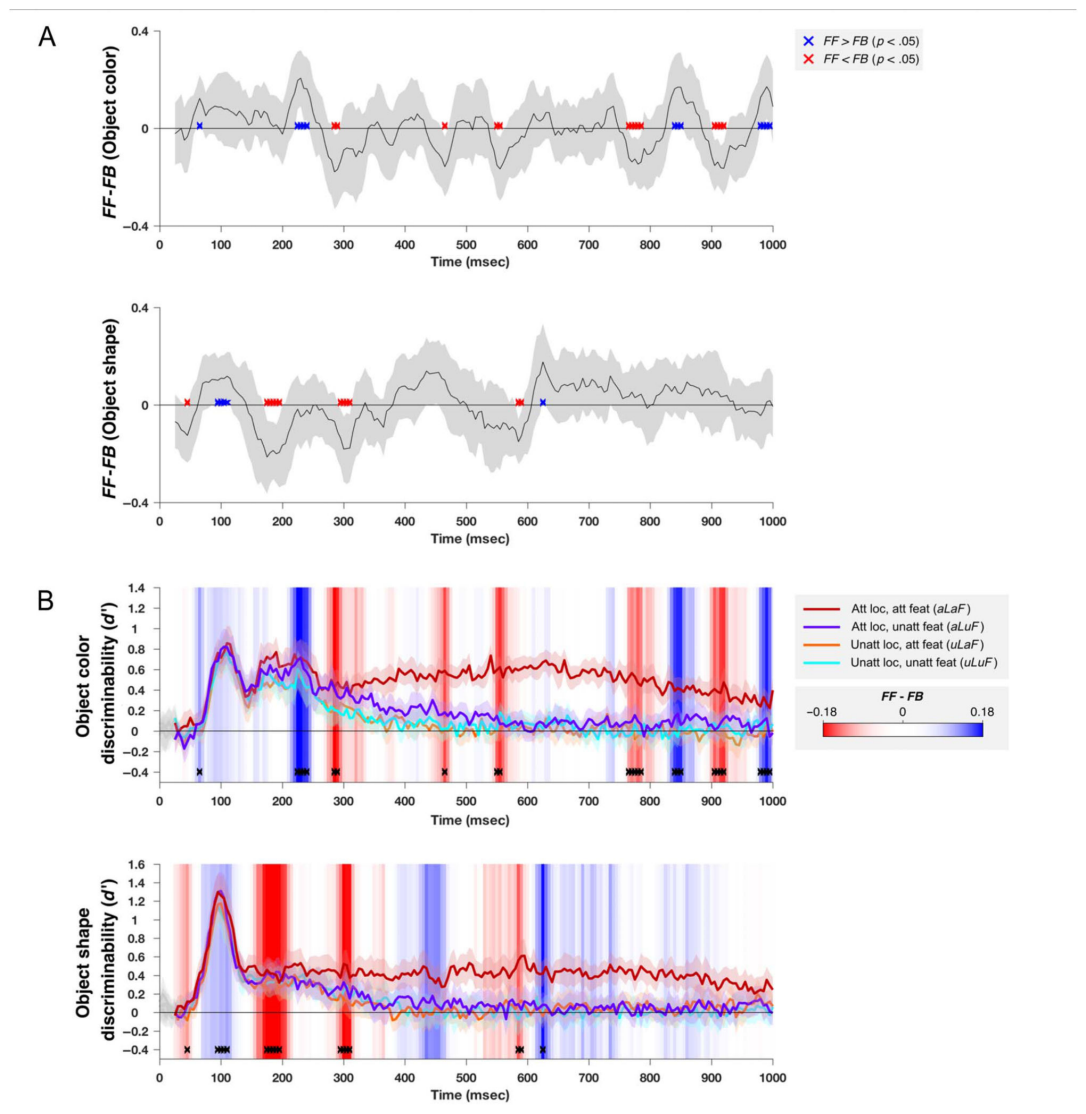
Analysis of feedforward and feedback interactions between occipital and frontal cortices. (A) *FF* (see Equation 3) minus *FB* (see Equation 4) based on classification performance on decoding stimulus color (top plot) and shape (bottom plot). Time samples at which the difference is significantly above or below zero (*FF* > *FB*, or *FF* < *FB*) are shown in blue and red, respectively (*p* values based on bootstrapped distribution, FDR corrected to *q* <.05). Shaded error bars indicate the 95% confidence interval of the between-subject mean. In (B), the occipital classification performance in each attention condition is replotted from Figure 4A. The background of the plot is colored according to the data from A, as indicated by the color bar. Time samples where *FF* - *FB* was significantly different from zero are also replotted, here with black crosses.

The results of this analysis are plotted in Figure 7. For both color and shape, the earliest time samples were dominated by feedforward information flow (*FF* > *FB*), consistent with the early visual responses in occipital cortex being relayed to frontal regions. These were followed by periods of feedback information flow, starting at 285 and 185 msec for color and shape, respectively. In both cases, information flow was biased toward the feedback direction until ~400 msec after stimulus onset. Interestingly, for both color and shape, the timing of the feedback information flows align with the onsets of the largest differences in stimulus decoding across attention condition, despite the later onset of these effect for color than for shape. This is seen in Figure 7B, where the large divergence between the dark red line (task-relevant information) and the other conditions starts around the onset of the first red region (*FB* > *FF*), for both color (top panel) and shape (bottom panel). This is compatible with the suggestion that frontal feedback to occipital regions drives the large attentional effects observed in occipital cortex after about 300 msec. Moreover, it suggests that the exchange of stimulus-related information, specifically, is important in driving the selection of attended information in occipital cortex.

Information exchange followed different time courses for color and shape information. For color, the early dominance of feedforward information persisted for longer (until 240 msec) than that for shape (until 115 msec). This extra period of feedforward information flow for color appears to correspond to the second early peak in decoding performance (~ 165−240 msec after stimulus onset) and could be related to higher order processing of color information by occipital cortex at this time, such as the ventral temporal occipital areas (Lafer-Sousa, Conway, & Kanwisher, 2016; Mullen, Dumoulin, McMahon, de Zubicaray, & Hess, 2007). Conversely, because the shape dimension we constructed for this study is highly artificial and unlikely to correspond to a feature dimension of relevance in the occipital cortex, it could be that the earlier feedback signal in this case is related to the frontal cortex’s involvement in storing information about the shape task and in modifying the responses of occipital areas in such a way that the object’s position along the shape dimension can be read out.

As with any correlation, it is possible that our partial correlations reflect correlation with another (untested) area. Therefore, although our results are consistent with a late dominance of feedback from frontal to occipital regions, it is possible that the feedback could originate in another area (e.g., parietal cortex; see Lauritzen, D’ Esposito, Heeger, & Silver, 2009). It is also possible that our source reconstruction did not accurately isolate frontal and occipital regions and that either of these includes signals from nearby regions. However, note that if, for example, any parietal signals were present in both frontal and occcipital ROIs, or in the unlikely event that frontal signals were present in the occipital ROI or vice versa, this would tend to reduce the measures of feedfoward and feedback information flows, rather than introduce false positives, making this a conservative analysis. Indeed, the presence of significant feedfoward and feedback information flows at all provides evidence that the ROIs were well segregated from one another, as does the absence of early classification performance in the frontal ROI.

## Discussion

We set out to address three open questions about the neural mechanisms supporting selective attention: the interaction between subtypes of attention on information coding, whether they induce similar or distinct effects on population tuning, and the dynamics of interregional information exchange giving rise to them. We found, first, that both spatial and feature-selective attention robustly boosted the stimulus-related information and that, when the effects of both were present, they interacted multiplicatively. Second, we found systematic differences in their pattern of enhancement across fine and coarse feature discriminations, which are consistent with differences from single-unit work in nonhuman primates. Third, through our information flow analysis of Granger causal relationships, we found evidence for the influence of frontal codes on occipital ones, with the onset of this influence coinciding with the onset of large attentional effects in occipital regions. We consider each of these findings below.

### Spatial and Feature-selective Attention Interact Multiplicatively to Enhance Coding of Relevant Stimulus Information

For the decoding of both color and shape, we found that spatial and feature-selective attention interacted multiplicatively, rather than having additive effects, resulting in a selective representation of task-relevant information. Additive effects are suggested by the integrated competition hypothesis of attention (Haazebroek et al., 2017; O’Craven et al., 1999; Duncan et al., 1997; Duncan, 1984), which predicts that both relevant and irrelevant features of an attended object will be boosted (object-based attention). Additive effects are also suggested by the empirical observation that feature-selective attention can sometimes modulate responses at unattended locations (e.g., Jehee et al., 2011; McAdams & Maunsell, 2000). However, in our data, when the effects of both types of attention were present, there was a clear multiplicative effect, with only the attended feature of the attended object prioritized and no advantage for unattended features of attended objects or attended features of unattended objects.

It has been suggested elsewhere that spatial and feature-based attention could combine additively in cases of low stimulus competition (when stimuli are dissimilar, e.g., McAdams & Maunsell, 2000), as well as in the earliest part of the stimulus-induced response to more similar stimuli, followed by multiplicative interactions later in the time course when stimuli compete to control the response (e.g., White, Rolfs, & Carrasco, 2015). This account seeks to reconcile the electrophysiological evidence of additive effects with psychophysical evidence of multiplicative interactions (e.g., White et al., 2015; Kingstone, 1992). In line with this possibility, we did observe an effect of spatial attention alone at earlier time points. Particularly for color, there appeared to be an advantage for representation of the unattended feature of the attended object (relative to features of the unattended object) in line with object-based accounts. However, the absence of feature-selective attention effects in this earliest stage of the response means that our data do not provide definitive evidence in favor of early additive effects. The effects of feature-selective attention emerged relatively late (from around 300 msec) and interacted with the effects of spatial attention, again consistent with a later emergence of multiplicative interactions. This late emergence of feature-selective attention effect might reasonably reflect processes associated with the maintenance of the relevant feature value in working memory. Previous fMRI (Serences, Ester, Vogel, & Awh, 2009) and ERP (Woodman & Vogel, 2008) studies demonstrate that relevant feature information is selectively maintained during working memory.

The absence of feature-selective attention effects in the earliest part of the response is consistent with previous reports of feature-selective attention effects that emerge after the initial transient response (Mirabella et al., 2007; Hillyard & Münte, 1984) or become stronger over time (Chen et al., 2012). There are also similar reports for feature-based attention where the earliest responses in occipital and frontal areas show little modulation (Bartsch et al., 2017; Bichot et al., 2015; Zhou & Desimone, 2011). However, there are conditions under which feature-based attention has been shown to modulate responses from the earliest onset (Zhang & Luck, 2009), which was not the case here.

### Differential Effects of Spatial and Feature-selective Attention

We found systematic differences between spatial and feature-selective attention in their patterns of enhancement across stimulus difference. For decoding of color (and to a lesser extent for shape), feature-selective attention produced a relatively greater enhancement of classifier performance for small physical differences than for large differences, as compared with the effects of spatial attention. We tested whether these differences in stimulus coding by the population might reflect differences propagated from the single-cell level by modeling population-level effects using a normalization model (Reynolds & Heeger, 2009). Normalization models of attention can account for a range of the effects of attention observed at the level of a single neuron (Ni & Maunsell, 2019; Ni, Ray, & Maunsell, 2012; Boynton, 2005, 2009; Lee & Maunsell, 2009; Reynolds & Heeger, 2009). Here, we adapted a normalization model to see if it could also capture attention-based changes in the information carried by the population response. Although the Reynolds and Heeger (2009) model is designed to model the effects of feature-based attention (attending to a feature value), feature-selective attention (attending to a feature-dimension) has been demonstrated to have similar effects at the level of single neurons (Cohen & Maunsell, 2011), and here we found that model predictions for feature-based attention captured the qualitative effects of feature-selective attention in our data.

Normalization models are based on the average effect of attention on the responses of single neurons, without modeling the heterogeneity of effects across neurons. Furthermore, attention can induce population-level changes beyond those reflected in individual tuning curves. One example is altering the correlation structure of a population response, which can significantly affect the information carried by the population (Verhoef & Maunsell, 2017; Sprague et al., 2015; Moreno-Bote et al., 2014; Cohen & Maunsell, 2009). Despite these simplifications, we found that a normalization model (Reynolds & Heeger, 2009) captured the different patterns of attentional enhancement in our data. Specifically, because the model predicts that feature-selective attention “sharpens” population tuning along the feature dimension, whereas spatial attention does not, it captured the main difference here that feature-selective attention produces a relatively greater enhancement of classifier performance for small physical differences than for large differences, as compared with the effects of spatial attention. In this way, our data show that these differences between spatial and feature-selective attention, demonstrated at a single-unit level, may also be the source of differences observed at the level of the population response in human cortex.

What does the success of the normalization model imply for how these features are coded by human cortex? We found the most marked difference between attention subtypes in the decoding of stimulus color in the occipital ROI. Of the two feature dimensions we manipulated (shape and color), it is more plausible for color that there are single units with response functions that approximate those in the normalization model. Neurons in a range of visual cortical areas are tuned for color (e.g., Hanazawa, Komatsu, & Murakami, 2000; Komatsu, Ideura, Kaji, & Yamane, 1992), and attention to color is a form of feature-based and feature-selective attention that has been investigated in single-unit work (Chen et al., 2012; Mirabella et al., 2007; Bichot et al., 2005; Motter, 1994). In contrast, the shape dimension is a more artificial, complex dimension, which could align with the feature selectivity of neurons in an area with intermediate to high-level shape selectivity, (e.g., V4; see Pasupathy, 2006) but is unlikely to correspond to a population code in the same way as for color. We found only subtle differences between spatial and feature-selective attention for the shape dimension, and these only reached significance at a few time points. However, where attention subtype differences were significant for shape (in the sensor-level decoding), the effect was in the same direction as for color, in line with the model predictions. This preliminary evidence suggests that a population tuning curve framework may also be helpful for understanding the effects of attention on arbitrary, higher level feature dimensions. It opens the possibility of using such models more generally as an explanatory bridge between the single-unit and population levels of description and further characterizing the similarities and differences between these levels of description. Further predictions of the model could also be tested at a population level, for instance, the prediction that spatial attention should induce more sharpening along the spatial dimension than feature-selective attention. When model parameters are further constrained by data, another direction for future work is to test quantitative as well as qualitative predictions of these models.

### Information Flow Analysis: The Role of Frontal Feedback in Attentional Modulation

The earliest occipital response was primarily stimulus-driven, showing little modulation with attention, but strong attention effects emerged after about 300 msec. Our information flow analysis suggests a key role for information exchange from frontal areas, with the pattern of stimulus-related information coding in occipital cortex Granger-caused by information coding in the frontal lobe shortly beforehand. Moreover, the onset of feedback dominating the flow of information corresponded to the time at which the occipital lobes showed a divergence between task-relevant and task-irrelevant information. For decoding color, where there was a second early peak in classifier performance, this period was later (285 msec) than for decoding shape (185 msec), but in both cases, it aligned with the time at which information processing in the occipital lobes became dominated by the task-relevant information.

Seminal theories of prefrontal function converge on the proposal that pFC implements cognitive control by affecting processing in more specialized cortices (Duncan, 2001; Miller & Cohen, 2001; Dehaene et al., 1998; Desimone & Duncan, 1995). For example, one prominent model (biased competition model; Duncan, 2006; Desimone & Duncan, 1995) proposes that feedback from the pFC biases local competition in more specialized cortices in favor of task-relevant information. However, these important proposals do not specify the nature or content of this feedback. Our data build on these proposals by suggesting a particular role for exchange of stimulus-related information. Because our information flow analysis specifically tracks the representation of stimuli, rather than simple activation, we can specify that selective processing in occipital cortex arises, at least in part, from feedback of stimulus-related information.

Our suggestion is consistent with work demonstrating that the responses of frontoparietal regions contain stimulus-related information (e.g., Freedman et al., 2001) that increases with spatial (Woolgar et al., 2015) and feature-selective (Jacksonetal., 2017) attention. Attentional effects on stimulus responses by rhesus monkey prefrontal cells emerge over a timescale that is broadly consistent with our results (Kadohisa et al., 2013), as is the observation that attentional effects in frontal cortices can precede those in sensory cortex (e.g., Lennert & Martinez-Trujillo, 2013). It goes beyond these observations, however, in specifying that—with particular time courses—frontal codes both result from and, in turn, influence representation in occipital cortex. This also goes beyond the proposal that frontal regions induce a preparatory biasing of sensory regions toward the attended content or a target template (see the review by Battistoni, Stein, & Peelen, 2017), because our results suggest that the feedback information contains stimulus information, rather than a purely attentional template. At the point that frontal codes dominantly exert (rather than receive) influence, selective processing begins to arise. This is consistent with an interactive system in which selective attention arises from the dynamic exchange of stimulus information favoring task-relevant processing.

Future work could build on these findings in two ways. First, because of the spatial uncertainty of MEG source reconstruction, we chose not to resolve into more fine-grained parcellations of the frontal lobe. However, this would be an interesting avenue for future work, ideally with concurrent EEG and individual MRI scans to help constrain the inverse problem. Second, with better source estimation, it would be interesting to examine the role of other brain regions, particularly the parietal lobe (which is known to have important roles in attention; e.g., Hebart, Bankson, Harel, Baker, & Cichy, 2018; Jerde, Merriam, Riggall, Hedges, & Curtis, 2012; Lennert et al., 2011; Woolgar, Hampshire, Thompson, & Duncan, 2011; Duncan, 2010). Our information flow analysis could be extended to a multiple regression framework (Kietzmann et al., 2019) to allow comparison between multiple regions.

## Conclusions

We found that both spatial and feature-selective attention enhanced the representation of visual information in human occipital and frontal cortices and that the two sub-types of attention interacted in a multiplicative way to yield selective processing of task-relevant stimulus aspects. We found differences in how spatial and feature-selective attention enhanced information across feature differences, which were consistent with modeling based on the distinct effects of spatial and feature-selective attention at the level of single cells. This suggests that changes in the tuning of single units may propagate to population-level coding, even if the latter is also affected by changes, for example, in correlation and covariance. An information flow analysis specified the dynamics of information exchange between occipital and frontal lobes and suggested that the largest attentional effects in occipital areas may be driven by feedback of stimulus-related information from frontal areas.

## Figures and Tables

**Table 1:** Model Parameters

Model Parameter	Parameter Description	Values Tested
*stimWidth*	Spatial extent of stimulus	25 (fixed value)
*stimFeatureWidth*	Extent of stimulus along feature dimension	25 (fixed value)
*ExWidth*	Spread of stimulation field along spatial dimension	30, 40, 50, 60, 70, 80, 90 or 100
*EthetaWidth*	Spread of stimulation field along feature dimension	30, 40, 50, 60, 70 or 80
*IxWidth*	Spread of suppressive field along spatial dimension	= C*ExWidth, where *C* = 1.5, 2, or 2.5
*IthetaWidth*	Spread of suppressive field along feature dimension	= C*EthetaWidth, where *C* = 1.5, 2, or 2.5
*AxWidth*	Extent/width of the spatial attention field	= *ExWidth*
*AthetaWidth*	Extent/width of the featural attention field	= *EthetaWidth*
*ApeakX*	Peak amplitude of spatial attention field	2, 4, 6, or 8
*ApeakTheta*	Peak amplitude of the feature-based attention field	2, 4, 6, or 8
*Abase*	Baseline of attention field for unattended locations/features	1 (fixed value)
*baselineMod*	Amount of baseline added to stimulus drive	0,.1,.3,.5, or 1
*baselineUnmod*	Amount of baseline added after normalization	0,.1,.3,.5, or 1
*sigma*	Constant that determines the semisaturation contrast	1e^-6^ (fixed value)
*Ashape*	either “oval” or “cross”	“oval” (fixed value)

Model parameters from the normalization model of attention (Reynolds & Heeger, 2009) that we used in model simulations. We defined the stimulus and response matrices as varying from -200 to 200 along both spatial and feature dimensions (arbitrary units). We generated the model predictions for every combination of the above model parameters, resulting in 172,800 sets of model predictions.

## Data Availability

All the raw data and the results of our classification analyses are available on an Open Science Framework project (https://doi.org/10.17605/OSF.IO/V893T).
